# Distribution and potential involvement of PTEN in the innate immune response during viral infections in Cherry Valley ducks

**DOI:** 10.3389/fimmu.2025.1570872

**Published:** 2025-05-20

**Authors:** Wende Chen, Shaojie Han, Rong Li, Shuo Li, Zhi Cao, Qing Pan, Gen Li

**Affiliations:** ^1^ College of Veterinary Medicine, Qingdao Agricultural University, Qingdao, Shandong, China; ^2^ Laboratory of Virology, Department of Translational Physiology, Infectiology and Public Health, Faculty of Veterinary Medicine, Ghent University, Merelbeke, Belgium

**Keywords:** PTEN, Cherry Valley duck, cloning, viral infection, innate immunity

## Abstract

**Introduction:**

Phosphatase and tensin homolog (PTEN) is a well-established tumor suppressor gene that antagonizes the PI3K/AKT signaling pathway and plays a critical role in regulating both innate and adaptive immune responses. However, its function in avian species, particularly in ducks, remains largely unexplored.

**Methods:**

In this study, the full-length cDNA of duck PTEN (*du*PTEN) was cloned from the spleen of healthy Cherry Valley ducks. Sequence alignment and phylogenetic analysis were performed to evaluate its structural conservation and evolutionary relationships. The tissue distribution of duPTEN mRNA was examined using quantitative real-time PCR (qRT-PCR). Furthermore, duPTEN expression was assessed in the spleen, lung, and brain at 1, 3, and 5 days post-infection (dpi) following exposure to Duck Tembusu Virus (DTMUV), Duck Plague Virus (DPV), and Novel Duck Reovirus (NDRV).

**Results:**

Sequence analysis demonstrated that *du*PTEN shares a high degree of conservation with PTEN from other avian species, displaying 100% identity with sequences from *Gallus gallus* and *Meleagris gallopavo*. qRT-PCR results revealed that *du*PTEN is ubiquitously expressed across multiple tissues, with the highest expression observed in the brain. Upon DTMUV and DPV infection, duPTEN expression was significantly upregulated in the spleen and lung but downregulated in the brain. In contrast, NDRV infection led to consistent downregulation of *du*PTEN across all three tissues.

**Discussion:**

This study is the first to characterize the molecular cloning, tissue-specific expression, and virus-induced regulation of *du*PTEN in ducks. The findings suggest that *du*PTEN plays a role in the host immune response to diverse viral infections, highlighting its potential involvement in the regulation of antiviral innate immunity in avian species.

## Introduction

As one of the most famous and frequently mutated tumor suppressor genes, the phosphatase and tensin homolog (PTEN) is mapped to human chromosome 10q23 (1–3). Its canonical role is to dephosphorylate phosphatidylinositol 3,4,5-trisphosphate (PIP3), thereby negatively regulating the PI3K pathway (4, 5).

The structure of PTEN is relatively simple, primarily consisting of two folded globular domains (a phosphatase domain (15–185 amino acids (aa)) and a C2 domain (192-353aa)) and a C-tail (carboxyl-terminal) (6). The phosphatase domain shares homology with tensin and auxilin, and contains a highly conserved phosphatase active site: HCxxGxxR. This domain can interact intramolecularly, thereby mediating the formation of PTEN dimers (7). The C2 domain of PTEN is structurally similar to the C2 domains of other phospholipases, such as Cδ1 and A2 phospholipases, and plays a role in regulating PTEN subcellular localization (8). In addition, the C2 domain is critical for PTEN dimers formation and interacts with phosphatase domain to regulate its phosphatase activity (7). The C-tail is mainly composed of some disordered segments, such as the PEST (Pro-Glu-Ser-Thr) sequences and PDZ domain binding motif (PDZ-BM) (9, 10). These disordered segments are key regulators of PTEN activity, stability, homo-dimer formation and post-translational modification (6, 11). Additionally, the eight residues ‘HTQITKVT’ at the PTEN C-terminal are essential for PDZ domain recognition, while phosphorylation of the Ser380/Thr382/Thr383 reduces PDZ domain binding (8, 12, 13). The dephosphorylation activity of PTEN is determined by the structure of the N-terminal phosphatase domain, but it is regulated collaboratively by the C2 and C-tail domains (14). It can be seen that the simplicity of PTEN’s structure cannot conceal the complexity of its regulation.

With increasing research on PTEN, it has become evident that its structure is growing more diverse and its activity is becoming increasingly complex. PTEN is located both in the cytoplasm and nucleus, and its optimal substrate is PIP3. One of its central functions is to control plasma membrane binding, as this is where the PIP3 substrate is located (6). To date, several translational variants of PTEN have been characterized, including PTEN-Long (PTEN-L) and PTEN-β (15–17). PTEN-L contains a 173-amino acid N-terminal extension (NTE) variant, translated from an upstream start site CUG (15). Similarly, PTEN-β includes a 146-amino acid amino-terminal extension variant, which is localized primarily in the nucleolus (17). Research has shown that the amino-terminal extensions of PTEN-L and PTEN-β may be disordered, but these extensions can affect the localization and activity of PTEN (18, 19).

Previous studies have identified a pivotal role for PTEN in the induction of type I interferon, a hallmark of antiviral innate immunity (20). PTEN is also capable of controlling the nuclear import of IRF3, a master transcription factor responsible for IFN-β production (20). These findings may just be the tip of the iceberg of PTEN’s antiviral innate immune function. As the world’s largest producer of meat ducks, China must undertake in-depth research on the immune system of ducks (21). In the present study, we cloned the CDs of the duck PTEN (*du*PTEN) and examined its tissue distribution. To further explore the antiviral capability of *du*PTEN, we investigated its response to various viruses. Our findings revealed that *du*PTEN plays a crucial role in regulating the inflammatory response and influencing the progression of viral infection.

## Materials and methods

### Animals and viruses

One-day-old Cherry Valley ducks were purchased and raised for three weeks. Before starting the experiment, enzyme-linked immunosorbent assays (ELISAs) were performed to confirm that the ducks were negative for Novel duck reovirus (NDRV), Duck Tembusu virus (DTMUV), and Duck Plague virus (DPV). All ducks used in this study were verified to be free of these viruses. The DTMUV, NDRV, and DPV strains used in this study were obtained from our laboratory and have been used in previous studies (22–24). The viral titers were determined using DEFs (duck embryo fibroblast) infection assays and calculated as the median tissue culture infective dose (TCID_50_)/mL, following the method of Reed and Muench (25).

One hundred and twenty 3-week-old ducks were randomly divided into four groups, with 30 ducks in each group. Three groups of Cherry Valley ducks were injected intramuscularly with DTMUV (10^5.2^ TCID_50_/mL, 0.4 mL per duck, containing 10^5.8^ TCID50 virus) (26), NDRV (10^4.5^ TCID_50_/mL, 0.5 mL per duck, containing 10^4.8^ TCID50 virus) (27), and DPV (10^6.5^ TCID_50_/mL, 0.3 mL per duck, containing 10^7.0^ TCID50 virus) (28), respectively. The control group was injected intramuscularly with 0.4 mL of phosphate-buffered saline (PBS). For PTEN tissue distribution analysis, three healthy Cherry Valley ducks were used. Additionally, the spleen, lung and brain of virus-infected ducks were collected at 1-, 3-, and 5-days post infection (dpi) for gene expression analysis. Over time, the clinical symptoms of infected ducks gradually decreased and returned to normal, the remaining ducks were euthanized by injecting lethal doses of pentobarbital sodium at 14 dpi.

### RNA extraction

Total RNA was extracted from heart, liver, spleen, lung, kidney, brain, cerebellum, brainstem, trachea, esophagus, proventriculus, gizzard, duodenum, jejunum, ileum, cecum, bursa of Fabricius, thymus, muscle and skin of 3-week-old Cherry Valley ducks using Trizol reagent (9108, Takara, Dalian, China). RNA was also extracted from the spleen, lung, and brain tissues of three randomly selected ducks from each of the four experimental groups at 1, 3, and 5 dpi using the same method. Reverse transcription of RNA sample to cDNA was performed using HiScript II One-Step RT-PCR kit (R223-01, Vazyme, Nanjing, China). The RNA concentration was determined by measuring absorbance at 260 nm, and RNA quality was confirmed by the A_260_/A_280_ ratio by Nanodrop 2000 Spectrophotometer (Thermo Fisher Scientific, Wilmington, DE, USA)

### Cloning of *du*PTEN

To obtain the coding sequences (CDs) of *du*PTEN, a set of specific polymerase chain reaction (PCR) primers was designed to identify the *du*PTEN sequences based on the predicated genes of chicken PTEN in the National Center for Biotechnology Information (NCBI) database (**Table 1**) (29). Total RNA (1 μg) was extracted from the spleen of healthy Cherry Valley ducks using TRIzol reagent (9108, Takara, Dalian, China), and cDNA was synthesized with the HiScript II One-Step RT-PCR kit (R223-01, Vazyme, Nanjing, China). The PCR conditions were as follows: an initial denaturation at 94°C for 5 minutes; followed by 35 cycles of denaturation at 94°C for 30 seconds, annealing at 57°C for 30 seconds, and extension at 72°C for 4 minutes; with a final extension at 72°C for 10 minutes. The full-length cDNA of *du*PTEN was sequenced by the Shanghai Invitrogen Biotechnology Co., Ltd. PCR products were visualized on 1% agarose gels and purified by using the agarose gel DNA fragment recovery kit (DP214-02, TIANGEN, Beijing, China). The resulting sequences were analyzed using Editseq software (DNAStar Lasergene, Version 18.0) (30).

**Table 1:** Primers used in this study.

Primer name	Sequence (5`– 3`)	Purpose
*du*PTEN-F	atgggttttcctgcagagag	Gene cloning
*du*PTEN-R	tcagacttttgtaatctgcg	
q-*du*PTEN-F	caattcccagtcagagacgct	qRT-PCR
q-*du*PTEN-R	tcttcacgtcttgagggtcc	
q-*du*β-actin-F qd β-actin R	ggtatcggcagcagtctta	qRT-PCR
q-*du*β-actin-R	ttcacagaggcgagtaactt	

F, forward primer; R, reverse primer; q, qRT-PCR.

### Phylogenetic analysis

The homology analysis of the *du*PTEN sequence was performed using the BLAST program of NCBI. The amino acid sequence of PTENs from various species were retrieved from NCBI, and the corresponding protein accession numbers are listed in **Table 2**. MegAlign software was used to analyze the similarity and evolutionary relationships of the PTEN amino acid sequence. The structure of the *du*PTEN amino acid sequences was predicted using the SMART online tool (http://smart.embl-heidelberg.de/). Multiple amino acid sequence alignments were performed using ClustalW2 (http://www.ebi.ac.uk/Tools/clustalw2/index.html) and further edited using the online tool Boxshade (http://www.ch.embnet.org/software/BOX_form.html). Phylogenetic analysis was generated using the MEGA 11.0 software, and the phylogenetic tree was constructed using the maximum- likelihood method.

**Table 2:** Reference species information of PTEN.

Species	GenBank accession numbers
Sus scrofa	NP 001137168.1
Vicugna pacos	XP 015096736.1
Saimiri boliviensis	XP 003922485.1
Papio anubis	XP 003904004.1
Macaca nemestrina	XP 011738414.1
Macaca mulatta	NP 001247894.1
Homo sapiens	NP 000305.3
Gorilla	XP 004049787.1
Felis catus	XP 003993913.1
Equus przewalskii	XP 008534286.1
Equus caballus	NP 001304189.1
Equus asinus	XP 014719844.1
Canis lupus familiaris	NP 001003192.1
Ailuropoda melanoleuca	XP 002914431.1
Oryctolagus cuniculus	XP 002718539.1
Loxodonta africana	XP 003409298.1
Bos taurus	XP 613125.4
Ovis aries	XP 011957880.1
Pantholops hodgsonii	XP 005982308.1
Mus musculus	NP 032986.1
Rattus norvegicus	NP 113794.1
Leptonychotes weddelli	XP 006732678.1
Chelonia mydas	XP 007071643.1
Pelodiscus sinensis	XP 014428208.1
Anser cygnoides	XP 013028245.1
Gallus gallus	XP 015134187.1
Meleagris gallopavo	XP 010712456.1
Geospiza fortis	XP 005430338.1
Taeniopygia guttata	XP 002186954.3
Oreochromis niloticus	XP 003449455.1

### Quantitative real time PCR

Total RNA from the above tissues was extracted and reverse-transcribed using the previously described method. Quantitative real-time PCR (qRT-PCR) primers of *du*PTEN were designed based on the *du*PTEN sequences obtained in this study, using Primer 3 software (http://www.broad.mit.edu/cgi-bin/primer/primer3, www.cgi), and were selected based on the dissociation curves. The expression of *du*PTEN was normalized to the endogenous reference gene of β-actin (**Table 1**). qRT-PCR was performed using the ChamQ™ SYBR^®^ qPCR Master Mix (Q711-02, Vazyme, Nanjing, China) on the 7500 Fast Real-Time PCR System (Applied Bio-systems, CA, USA). The PCR reaction volume was 20 μL, and the conditions were as follows: an initial denaturation at 95°C for 5 minutes, followed by 40 cycles of denaturation at 95°C for 10 seconds and extension at 60°C for 34 seconds. A dissociation curve analysis was performed at the end of the reaction. Each sample was analyzed in triplicate.

### Calculations and statistical analysis

The relative expression levels of the *du*PTEN were measured using the duck β-actin gene as the endogenous reference gene and calculated using the 2^−ΔΔCt^ method (31). All data were represented as mean ± SD of triplicate samples and analyzed by using SPSS19.0 software. Graphs were generated with Graph Pad Prism 5.0 software (Graph Pad Software Inc., San Diego, CA, USA). Statistical differences were assessed using one-way ANOVA followed by Dunnett’s multiple comparisons test. Significant and highly significant differences were set at *P* < 0.05 and *P* < 0.01, respectively.

## Results

### Molecular characterization of *du*PTEN

**Figure 1:**
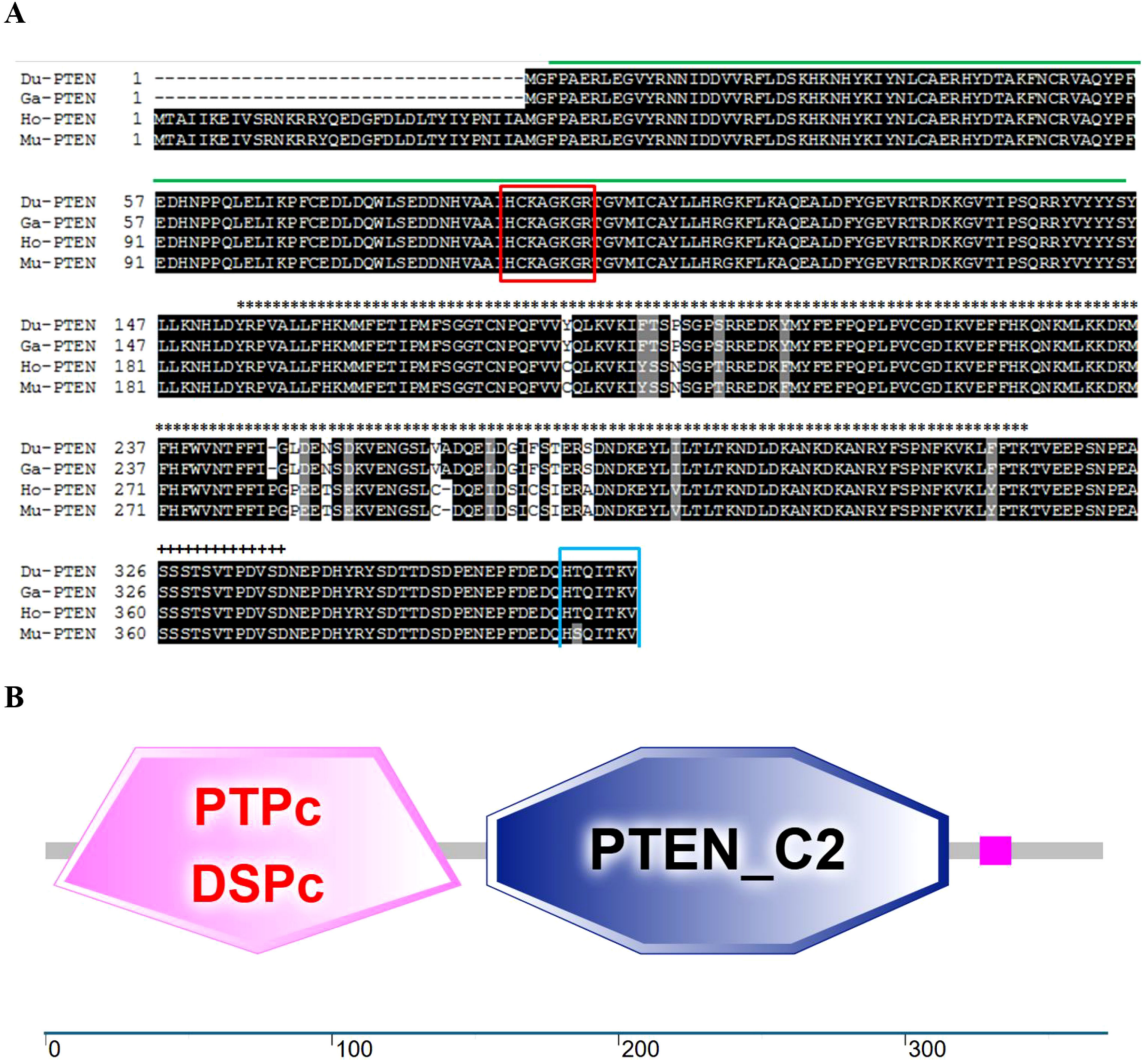
Characterization of *du*PTEN. **(A)** Alignment of the deduced AA sequence of *du*PTEN with other species. Black shading indicates AA identity; gray shading indicates similarity (50% threshold). The green line represents the PTPc-DSPc domain; the∗∗∗above the sequence represents the C2 domains; the+++above the sequence represents the low complexity domains. The red box is the phosphatase active site: HCxxGxxR, and the blue box is the C-terminal eight residues. **(B)** Prediction of *du*PTEN protein domains by the SMART program. *Du*PTEN contains the PTPc-DSPc domain (3-145aa), and the C2 domains (154-315aa), and the low complexity domains (326-337aa). Du, Cherry Valley Duck, Ga, Gallus gallus; Ho, Homo sapiens, Mu, Mus musculus.

The complete open reading frame of *du*PTEN was 1,110bp in length, encoding a protein of 369 aa. The sequence has been submitted to GenBank (GeneBank accession number: PV054948). Our results indicated that the *du*PTEN contains the typical phosphatase active site ‘HCKAGKGR’ (highlighted in the red box, **Figure 1A**). Additionally, the C-terminal includes seven residues, ‘HTQITKV’ (highlighted in the blue box, **Figure 1A**), which maybe critical for specific recognition of the PDZ domain. Secondary structure prediction using the SMART program revealed that *du*PTEN possess three characteristic domains: the PTPc-DSPc domain (3-145aa, indicated by the green line), the C2 domains (154-315aa, marked with ∗∗∗ above the sequence), and a low-complexity domains (326-337aa, marked with +++ above the sequence) (**Figures 1A, B**).

### Phylogenetic analysis of *du*PTEN

**Figure 2:**
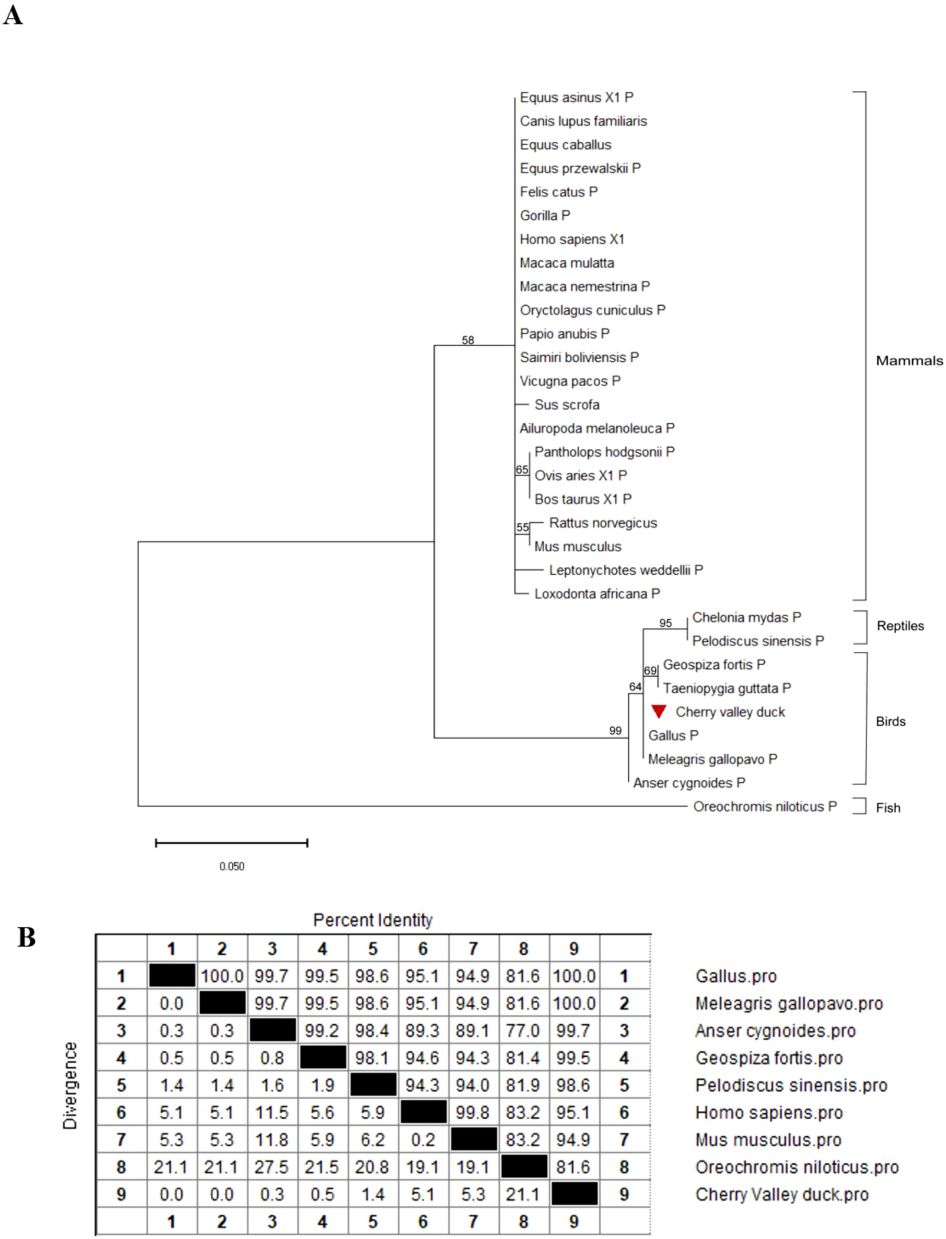
Phylogenetic analysis and sequence similarity of PTEN. **(A)** The phylogenetic tree of the AA sequence of *du*PTEN and other species. A Maximum Likelihood tree was generated using MEGA 11.0, and a 1,000-bootstrap analysis was performed to assess the reliability of the tree. The scale bar is 0.050. GenBank accession numbers are shown in **Table 2**. **(B)** Sequence similarity analysis of PTEN among different species. The program was performed using the MegAlign software.

To confirm the evolutionary relationship of *du*PTEN, a phylogenetic tree was constructed using the amino acid (AA) sequences of *du*PTEN and other PTENs, as shown in **Figure 2A**. The phylogenetic tree revealed that these PTENs sequences clustered into four majors’ branches: mammals, reptiles, birds and fish. *Du*PTEN was classified within the avian branch, showing a particular relationship with the PTEN of *Gallus* and *Meleagris gallopavo*, while being relatively distant from *Fish* PTEN. These results indicated that *du*PTEN shares a closer evolutionary relationship with avian PTENs. Further analysis of *du*PTEN involved comparing its AA sequence with those from birds, fish, mammals, and reptiles. The multiple sequence alignment showed that *du*PTEN shared 100% identity with the PTENs of *Gallus* and *Meleagris gallopavo*, over 90% identity with PTENs from mammals, birds, and reptiles, and 81.6% identity with *Oreochromis niloticus* (**Figure 2B**). These results highlight the strong conservation of PTEN across most species (**Figure 2B**).

### Tissue distribution of *du*PTEN in healthy Cherry Valley duck

**Figure 3:**
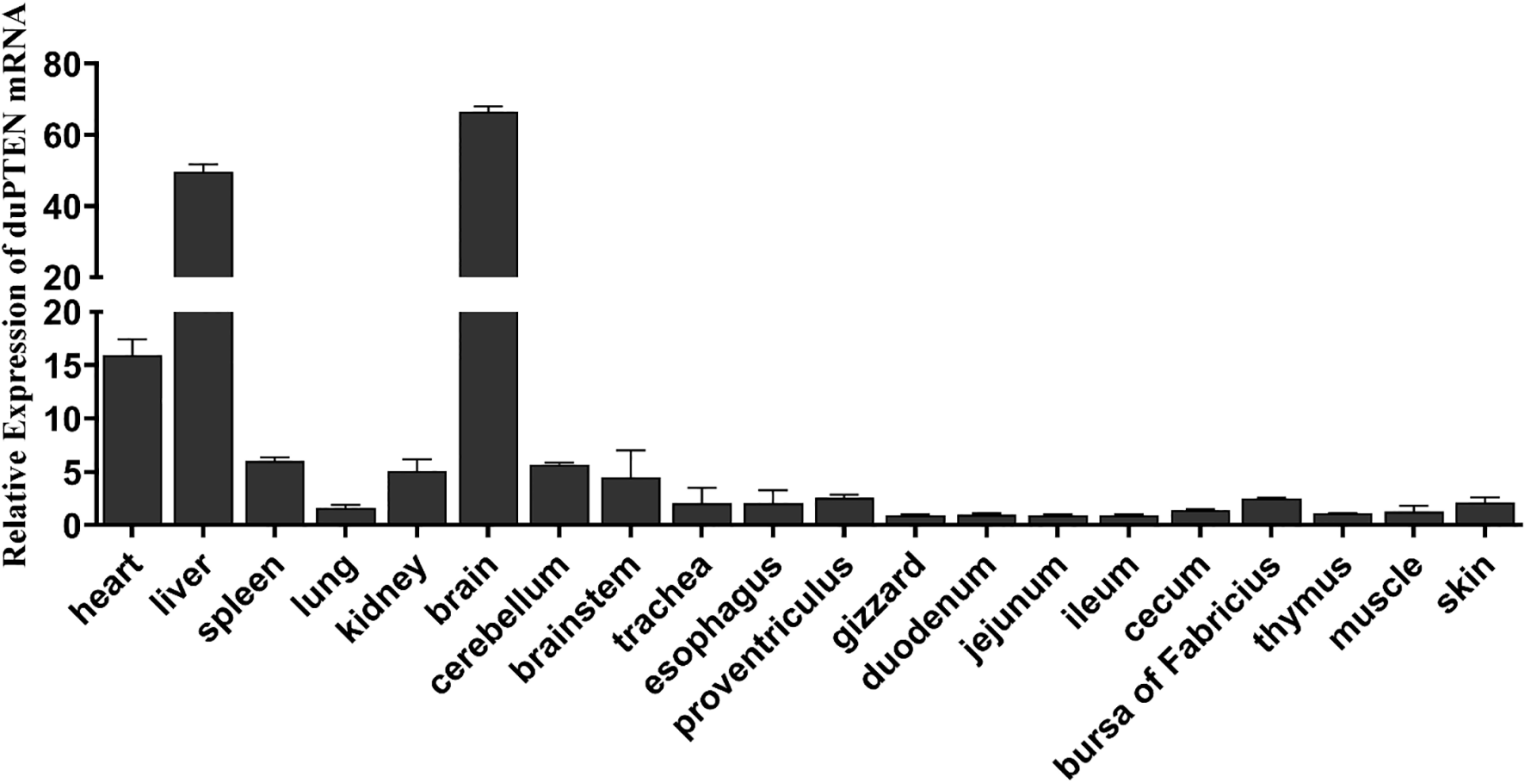
Tissue distributions of PTEN in the healthy Cherry Valley duck. The relative mRNA levels were normalized to the expression of the β-actin gene from various tissues, with data being normalized to the thymus. The relative expression levels of *du*PTEN were calculated using β-actin as the reference gene and analyzed using the 2^−ΔΔCt^ method. Means ± standard deviation from three independent repetitions are presented.

To analyze the expression levels of *du*PTEN mRNA in the tissues of healthy Cherry Valley ducks, three healthy ducks were randomly selected, and 20 tissue samples were collected. The thymus was used as the reference tissue. As shown in **Figure 3**, *du*PTEN mRNA expression was highest in the brain, followed by the liver and heart. In contrast, lower expression levels were observed in intestinal tissues, including the ileum, jejunum, and duodenum. The widespread expression of *du*PTEN indicates that the *du*PTEN might be extensively involved in the host immune response of healthy Cherry Valley ducks.

### Expression profiles of *du*PTEN in the viral infected ducks

**Figure 4:**
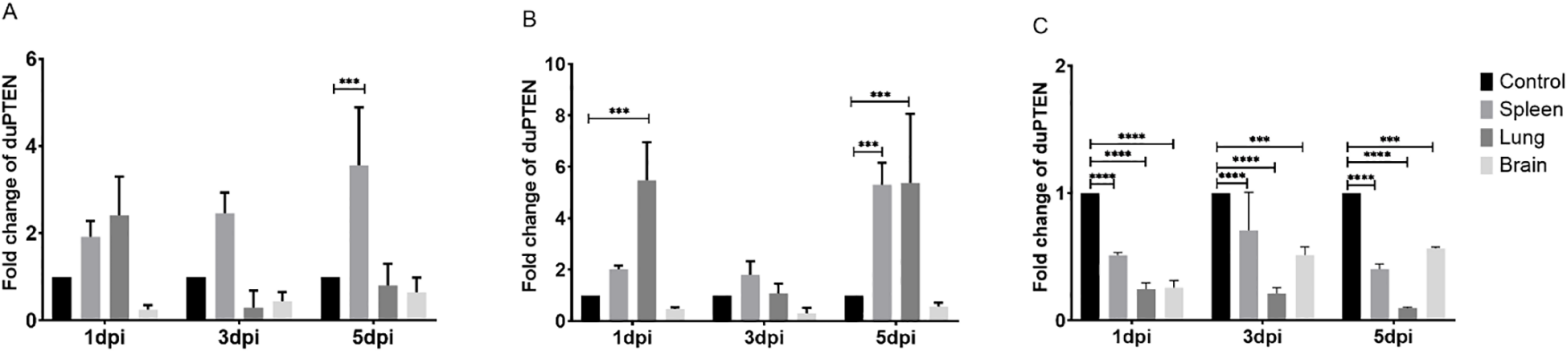
Analysis of duPTEN transcript at early stages of viral infection. **(A)** Expression Profiles of *du*PTEN in the DTMUV Infected Ducks, **(B)** Expression Profiles of *du*PTEN in the DPV Infected Ducks, **(C)** Expression Profiles of *du*PTEN in the NDRV Infected Ducks. The relative expression levels were calculated with the 2^−ΔΔCt^ method. Statistical significance was evaluated by one-way ANOVA followed by Dunnett’s multiple comparisons. Bar represents the mean ± standard deviation (n=3). *** (0.0001 ≤ *P* < 0.001), ****(*P* < 0.0001).

To determine the potential involvement of *du*PTEN in the host’s antiviral immune response against various viral infections, the mRNA expression levels of *du*PTEN were assessed in the spleen, lung, and brain, which represent key target, immune, and nervous system organs, respectively, during viral infection (26–28). Following infection with the three viruses, *du*PTEN expression was significantly decreased in the brain at 1, 3, and 5 dpi (**Figures 4A–C**), with the most pronounced downregulation observed in the NDRV-infected group (**Figure 4C**). In the spleen, *du*PTEN expression was upregulated during DTMUV and DPV infection, peaking at 5 dpi with 5.3-fold increase in the DPV-infected groups (*P* < 0.0005; **Figure 4B**). In the lung, *du*PTEN expression was significantly upregulated at the indicated time points in both DTMUV-and DPV-infected ducks (**Figures 4A, B**). Notably, *du*PTEN expression was consistently downregulated at all the time points and in all tissues during NDRV-infection group (**Figure 4C**). These findings suggest that *du*PTEN plays a role in the host’s immune response to multiple viral infections.

## Discussion

Discovered over 20 years ago as a “candidate tumor suppressor gene”, PTEN is an enzyme that is strictly regulated and capable of dephosphorylating both protein and lipid substrates (4). It plays a crucial role in various cellular processes, including proliferation, cell migration, apoptosis, cell survival, and metabolism. Additionally, PTEN is involved in numerous immunological processes and functions as a pro-inflammatory factor (5, 32).

In this study, PTEN was identified, cloned, and characterized for the first time from Cherry Valley ducks. The *du*PTEN contains a 1,110bp ORF and encodes three characteristic structure domains: the PTPc-DSPc domain (3-145aa), the C2 domains (154-315aa), and the low complexity domains (326-337aa). The PTPc-DSPc domain, which functions as the phosphatase domain, includes the highly conserved phosphatase active site motif HCxxGxxR (6, 33). Our finding shows that this structural motif in *du*PTEN is identical to that of *Homo sapiens*, *Gallus gallus* and *Mus musculus*, all of which feature the sequence ‘HCKAGKGR’. A key regulatory interface of PTEN is the interaction between the C2 domain and phosphatase domain (34). Unlike what Chen L et al. mentioned (8), our study found that the C-terminus of *du*PTEN only contains 7 residues, namely “HTQITKV”, and from our **Figure 1A**, it can also be seen that there are also only 7 residues “HTQITKV” in PTEN of species such as *Gallus*, *Homo*, and *Mus musculus*. Phylogenetic analysis revealed that *du*PTEN shares 100% identity with PTEN from *Gallus gallus* and *Meleagris gallopavo*, 95.1% identity with *Homo sapiens*, and 81.6% identity with *Oreochromis niloticus*, a finding consistent with the phylogenetic tree results. These data underscore the high conservation of PTEN structure and the homology of *du*PTEN across species.

Given the important role of PTEN in immune response, studying its tissue distribution can provide valuable insights into its functions and regulatory mechanisms. In our study, *du*PTEN was expressed in all tested tissues. Considering that PTEN role as a key regulatory factor in cellular growth and proliferation, its widespread expression across tissues is not surprising. The highest *du*PTEN expression was observed in the brain, followed by the liver and heart, while the lower expressions were detected in intestinal tissues such as ileum, jejunum and duodenum. Our previous research has shown that the same gene can exhibit different tissue distributions at various developmental stages and in different organs of the same animal. Further research is needed to elucidate the detailed biological function associated with the tissue distribution of *du*PTEN. Nonetheless, its broad expression indicates that the *du*PTEN might be extensively involved in the immune response of healthy Cherry Valley ducks.

Increasing evidence indicates that, beyond its well-established role in tumor suppression, PTEN plays a crucial role in IFN responses and antiviral innate immunity (8, 20). For example, PTEN deficiency in mouse prostate cancer cells leads to significant viral proliferation and cell lysis (35). Partially PTEN-deficient mice are more susceptible to vesicular stomatitis virus infection than wild-type (WT) mice (20). Similarly, PTEN inhibition in a murine model of sepsis results in increased inflammation, tissue damage, and mortality (36). Moreover, PTEN-L has been shown to promote type I IFN response and antiviral innate immunity in a phosphatase activity-dependent manner during viral infection (4). In this study, the antiviral role of *du*PTEN was further studied following infection with DTMUV, DPV and NDRV. Immune-related organs and the virus target tissues were selected for analysis. Significant changes in *du*PTEN expression levels were observed after infection with all three viruses. In the DTMUV- and DPV-infected groups, *du*PTEN displayed similar expression patterns: it was upregulated in the spleen and lung but downregulated in the brain. Considering that the brain is an important site for neurological symptoms, its widespread downregulation deserves further investigation. In contrast, *du*PTEN expression was consistently downregulated across all time points and tissues during NDRV infection. The spleen as the largest secondary lymphatic organ of birds, the *du*PTEN expression peaked at 5 dpi in the DTMUV- and DPV-infected groups. In DPV-infected lungs, *du*PTEN was significantly upregulated at 1 dpi and remained elevated through 5 dpi, indicating that lung-expressed *du*PTEN actively participate in the antiviral immune response. Similar upregulation of PTEN has been reported in B cells during acute and chronic human immunodeficiency virus infection (37, 38). However, in the brain, which exhibits the highest expression in healthy adult Cherry Valley duck, *du*PTEN was downregulation at all detection time points (1, 3, and 5 dpi) following infection with all three viruses. Previous studies have shown that PTEN deletion leads to a weakened inflammatory response (8). The observed alterations in *du*PTEN expression across different tissues and viruses suggest that viral infections disrupt the baseline expression of *du*PTEN *in vivo*. The distinct expression patterns may be attributed to variations in viral properties and activation pathways. Many viruses, such as influenza, have been shown to manipulate the PI3K/AKT pathway to promote viral replication and evade immune surveillance. The potential of PTEN to regulate immune responses through its interaction with the PI3K signaling pathway is an interesting aspect, and we plan to further explore this in future studies (39). The subcellular localization of PTEN is crucial for its antiviral function, as its presence in specific cellular compartments, such as the plasma membrane or mitochondria, modulates the PI3K/AKT signaling pathway to regulate immune responses. Further research will investigate the subcellular localization of PTEN during viral infection using western blot and immunofluorescence staining. Overall, these findings indicate that *du*PTEN plays a critical role in the host immune response to multiple viruses, likely through complex regulatory mechanisms within signaling pathways.

While the role and mechanisms of PTEN in tumor suppression have been widely studied, its involvement in adaptive and innate immunity is only beginning to be uncovered (3, 8). In this study, *du*PTEN was identified, and its function was analyzed. The expression of *du*PTEN in healthy Cherry Valley ducks was found to be widespread. Moreover, its relative expression levels changed significantly during viral infections *in vivo*. Together, our research enhances the understanding of *du*PTEN’s structure and role in antiviral responses, providing a foundation for further exploration of the function and regulatory mechanisms of PTEN in waterfowl.

## Data Availability

The datasets presented in this study can be found in online repositories. The names of the repository/repositories and accession number(s) can be found in the article/supplementary material.

## References

[1] Li J Yen C Liaw D Podsypanina K Bose S Wang SI. PTEN, a putative protein tyrosine phosphatase gene mutated in human brain, breast, and prostate cancer. Science 1997 28(3);. (5308) 275:1943–7. doi: 10.1126/science.275.5308.1943 9072974

[2] Steck PA Pershouse MA Jasser SA Yung WKA Lin H Ligon AH. Identification of a candidate tumour suppressor gene, MMAC1, at chromosome 10q23.3 that is mutated in multiple advanced cancers. Nat Genet. (1997) 15:356–62. doi: 10.1038/ng0497-356 9090379

[3] Taylor H Laurence ADJ Uhlig HH. The role of PTEN in innate and adaptive immunity. ld Spring Harbor Perspect Med. (2019) 2:9(12):a036996. doi: 10.1101/cshperspect.a036996 PMC6886458 31501268

[4] Cao Y Wang H Yang L Zhang Z Li C Yuan X. PEN-L promotes type I interferon responses and antiviral immunity. Cell Mol Immunol. (2018) 15:48–57. doi: 10.1038/cmi.2017.102 29057971 PMC5827174

[5] Pereiro P Figueras A Novoa B. Zebrafish pten Genes Play Relevant but Distinct Roles in Antiviral Immunity. Vaccines. (2020) 8(2). doi: 10.3390/vaccines8020199 PMC7349019 32357549

[6] Masson GR Williams RL. Structural mechanisms of PTEN regulation. Cold Spring Harbor Perspect Med. (2020) 2:a036152. doi: 10.1101/cshperspect.a036152 PMC7050585 31636093

[7] Lee JO Yang H Georgescu MM Di Cristofano A Maehama T Shi Y. Crystal structure of the PTEN tumor suppressor: implications for its phosphoinositide phosphatase activity and membrane association. Cell. (199) , 29:323–34. doi: 10.1016/s0092-8674(00)81663-3 10555148

[8] Chen L Guo D. The functions of tumor suppressor PTEN in innate and adaptive immunity. Cell Mol Immunol. (2017) 14:581–9. doi: 10.1038/cmi.2017.30 PMC5520418 28603282

[9] Georgescu MM Kirsch KH Kaloudis P Yang H Pavletich NP Hanafusa H. Stabilization and Productive Positioning Roles of the C2 Domain of Pten Tumor Suppressor. Cancer Res. (2000) 60(24):7033–8. 11156408

[10] Georgescu MM Kirsch KH Akagi T Hanafusa H. The tumor-suppressor activity of PTEN is regulated by its carboxyl-terminal region. Proc Natl Acad Sci. (1999) 31:10182–7. doi: 10.1073/pnas.96.18.10182 PMC17863 10468583

[11] Heinrich F Chakravarthy S Nanda H Papa A Pandolfi PP AH R. The PTEN tumor suppressor forms homodimers in solution. Structure. (2015) 23:1952–7. doi: 10.1016/j.str.2015.07.012 PMC4598300 26299948

[12] Takahashi Y Morales FC Kreimann EL Georgescu MM. PTEN tumor suppressor associates with NHERF proteins to attenuate PDGF receptor signaling. EMBO J. (2006) 25:910–20. doi: 10.1038/sj.emboj.7600979 PMC1383560 16456542

[13] Jurado S Benoist M Lario A Knafo S Petrok CN Esteban JA. PTEN is Recruited to the Postsynaptic Terminal for NMDA Receptor-dependent Long-term Depression. EMBO J. (2010) 18:2827–40. doi: 10.1038/emboj.2010.160 PMC2924645 20628354

[14] Al-Khouri AM Ma Y Togo SH Williams S Mustelin T. Cooperative phosphorylation of the tumor suppressor phosphatase and tensin homologue (PTEN) by casein kinases and glycogen synthase kinase 3β. J Biol Chem. (2005) 21:35195–202. doi: 10.1074/jbc.M503045200 16107342

[15] B. D Hopkins B Fine N Steinbach M Dendy ZA. Secreted PTEN phosphatase that enters cells to alter signaling and survival. Science. (2013) 26:399–402. doi: 10.1126/science.1234907 PMC3935617 23744781

[16] Liang H He S Yang J Jia X Wang P Chen X. PTENα, a PTEN isoform translated through alternative initiation, regulates mitochondrial function and energy metabolism. Cell Metab. (2014) 6:836–48. doi: 10.1016/j.cmet.2014.03.023 PMC4097321 24768297

[17] Liang H Chen X Yin Q Ruan D Zhao X Zhang C. PTENβ is an alternatively translated isoform of PTEN that regulates rDNA transcription. Nat Commun. (2017) 23:8:14771. doi: 10.1038/ncomms14771 PMC5376652 28332494

[18] Malaney P Pathak RR Xue B Uversky VN Davé V. Intrinsic disorder in PTEN and its interactome confers structural plasticity and functional versatility. Sci Rep. (2013) 3:2035. doi: 10.1038/srep02035 23783762 PMC3687229

[19] Masson GR Perisic O Burke JE Williams RL. The intrinsically disordered tails of PTEN and PTEN-L have distinct roles in regulating substrate specificity and membrane activity. Biochem J. (2016) 473:135–44. doi: 10.1042/BJ20150931 PMC4700475 26527737

[20] Li S Zhu M Pan R Fang T Cao YY Chen S. The tumor suppressor PTEN has a critical role in antiviral innate immunity. Nat Immunol. (2016) 17:241–9. doi: 10.1038/ni.3311 26692175

[21] Mi J Wang H Chen X Hartcher K Wang Y Wu Y. Lack of access to an open water source for bathing inhibited the development of the preen gland and preening behavior in sanshui white ducks. Poultry Sci. (2020) 99:5214–21. doi: 10.1016/j.psj.2020.08.018 PMC7647854 33142437

[22] Li Z Cai Y Liang G El-Ashram S Mei M Huang W. Detection of novel duck reovirus (NDRV) using visual reverse transcription loop-mediated isothermal amplification (RT-LAMP). Sci Rep. (2018) 8(1):14039. doi: 10.1038/s41598-018-32473-4 30232402 PMC6145877

[23] Li N Hong T Li R Wang Y Guo M Cao Z. Cherry valley ducks mitochondrial antiviral-signaling protein-mediated signaling pathway and antiviral activity research. Front Immunol. (2016) 21:377. doi: 10.3389/fimmu.2016.00377 PMC5030477 27708647

[24] Yan P Zhao Y Zhang X Xu D Dai X Teng Q. An infectious disease of ducks caused by a newly emerged tembusu virus strain in mainland China. Virology. (2011) 417:1–8. doi: 10.1016/j.virol.2011.06.003 21722935

[25] Reed LJ Muench H. A simple method of estimating fifty percent endpoints. Am J Trop Med Hygiene. (1938) 27:493–7.

[26] Li N Wang Y Li R Liu J Zhang J Cai Y. Immune responses of ducks infected with duck tembusu virus. Front Microbiol. (2015) 8:425. doi: 10.3389/fmicb.2015.00425 PMC4424876 26005441

[27] Li N Hong T Wang Y Wang Y Yu K Cai Y. The pathogenicity of novel duck reovirus in cherry valley ducks. Veterinary Microbiol. (2016) 30:192:181–185. doi: 10.1016/j.vetmic.2016.07.015 27527781

[28] Li N Hong T Li R Guo M Wang Y Zhang J. Pathogenicity of duck plague and innate immune responses of the cherry valley ducks to duck plague virus. Sci Rep. (2016) 24:6:32183. doi: 10.1038/srep32183 PMC4995378 27553496

[29] Yu Y Li L Sun R Xu Z Wang Q Ou C. Tissue distribution and developmental changes of PTEN in the immune organs of chicken and effect of IBDV infection on it. Poultry Sci. (2021) 100:101356. doi: 10.1016/j.psj.2021.101356 34358959 PMC8350381

[30] Burland TG. DNASTAR’s lasergene sequence analysis software. . Methods Mol Biol. (2000) 132:71–91. doi: 10.1385/1-59259-192-2:71 10547832

[31] Livak KJ Schmittgen TD. Analysis of relative gene expression data using real-time quantitative PCR and the 2(-delta delta C(T)) method. Methods. (2001) 25:402–8. doi: 10.1006/meth.2001.1262 11846609

[32] Sahin E Haubenwallner S Kuttke M Kollmann I Halfmann A Dohnal AM. Macrophage PTEN regulates expression and secretion of arginase I modulating innate and adaptive immune responses. J Immunol. (2014) 15:1717–27. doi: 10.4049/jimmunol.1302167 PMC4120896 25015834

[33] Patterson KI Brummer T O’Brien PM Daly RJ. Dual-specificity phosphatases: critical regulators with diverse cellular targets. Biochem J. (2009) 15:475–89. doi: 10.1042/bj20082234 19228121

[34] Haynie DT Xue B. Superdomains in the protein structure hierarchy: the case of PTP-C2. Protein Sci A Publ Protein Soc. (2015) 24:874–82. doi: 10.1002/pro.2664 PMC4420535 25694109

[35] Moussavi M Fazli L Tearle H Guo Y Cox M Bell J. Oncolysis of prostate cancers induced by vesicular stomatitis virus in PTEN knockout mice. Cancer Res. (2010) 15:1367–76. doi: 10.1158/0008-5472.CAN-09-2377 20145134

[36] Sisti F Wang S Brandt SL Glosson-Byers N Serezani CH. Nuclear PTEN enhances the maturation of a microRNA regulon to limit myD88-dependent susceptibility to sepsis. Sci Signaling. (2018) 11:eaai9085. doi: 10.1126/scisignal.aai9085 PMC8147521 29717063

[37] Getahu A Wemlinge SM Rudra P Santiago ML van Dyk LF Cambier JC. Impaired B Cell Function during Viral Infections due to PTEN-mediated Inhibition of the PI3K Pathway. J Exp Med. (2017) 214:931–41. doi: 10.1084/jem.20160972 PMC5379973 28341640

[38] de Armas LR Pallikkuth S Pan L Rinaldi S Cotugno N Andrews S. Single cell profiling reveals PTEN overexpression in influenza-specific B cells in aging HIV-infected individuals on anti-retroviral therapy. Sci Rep. (2019) 21:2482. doi: 10.1038/s41598-019-38906-y PMC6385500 30792481

[39] Ayllon J García-Sastre A Hale BG. Influenza A viruses and PI3K: are there time, place and manner restrictions? Virulence. (2012) 3:411–4. doi: 10.4161/viru.20932 PMC3478246 22722241

